# Progress in Nanotechnology Based Approaches to Enhance the Potential of Chemopreventive Agents

**DOI:** 10.3390/cancers3010428

**Published:** 2011-01-21

**Authors:** Irfana Muqbil, Ashiq Masood, Fazlul H. Sarkar, Ramzi M. Mohammad, Asfar S. Azmi

**Affiliations:** 1 Department of Biochemistry, Faculty of Life Sciences, Aligarh Muslim University, Aligarh 202002, UP, India; 2 Department of Oncology, Karmanos Cancer Institute, Wayne State University, Detroit, MI 48201, USA; 3 Department of Pathology, Wayne State University School of Medicine, Wayne State University, Detroit, MI 48201, USA

**Keywords:** chemoprevention, nanotechnology, nanochemoprevention

## Abstract

Cancer chemoprevention is defined as the use of natural agents to suppress, reverse or prevent the carcinogenic process from turning into aggressive cancer. Over the last two decades, multiple natural dietary compounds with diverse chemical structures such flavonoids, tannins, curcumins and polyphenols have been proposed as chemopreventive agents. These agents have proven excellent anticancer potential in the laboratory setting, however, the observed effects *in vitro* do not translate in clinic where they fail to live up to their expectations. Among the various reasons for this discrepancy include inefficient systemic delivery and robust bioavailability. To overcome this barrier, researchers have focused towards coupling these agents with nano based encapsulation technology that in principle will enhance bioavailability and ultimately benefit clinical outcome. The last decade has witnessed rapid advancement in the development of nanochemopreventive technology with emergence of many nano encapsulated formulations of different dietary anticancer agents. This review summarizes the most up-to-date knowledge on the studies performed in nanochemoprevention, their proposed use in the clinic and future directions in which this field is heading. As the knowledge of the dynamics of nano encapsulation evolves, it is expected that researchers will bring forward newer and far more superior nanochemopreventive agents that may become standard drugs for different cancers.

## 1. Introduction

The concept of chemoprevention gained momentum when numerous laboratory studies showed anticancer effects of natural agents in various tumor models [1]. However, natural agents failed to prove their worth at the last and most expensive step, *i.e.*, in the clinic [2]. Many reasons have been attributed to this failure that range from lack of clearly defined mechanism of action to poor bioavailability [3]. Further, the observed anti-cancer activity of different natural agents in cell culture and animal models does not translate into clinically beneficial outcome in humans. This is because laboratory studies tend to investigate pharmacological effects at doses that are not achievable in humans through dietary sources [4]. Based on these important and limiting factors the science of chemoprevention has less proponents and more critiques. Nevertheless since, the term chemoprevention was coined 40 years ago [5,6], researchers through extensive high throughput screening have identified an arsenal of anticancer drugs from natural sources [7]. The fleet is led by the flagship drug Taxol, which has been approved by the FDA for the treatment of several human malignancies [8]. Many other drugs originally discovered from nature have also been approved by the FDA, including camptothecin and its analogs (topotecan and irinotecan), vinblastine and vincristine, and the microbial-derived anthracyclines, such as doxorubicin and the bleomycins [9-13]. Several other promising compounds are currently being tested in clinical trials against cancer and other deadly chronic diseases.

Compounds such as resveratrol [14-16], curcumin [17,18], thymoquinone [19-21] and epigallocatechin (EGCG) have shown potent anticancer activity in cell culture and animal tumor models [22-28]. These chemopreventive agents have also demonstrated some clinical benefit in combination with standard chemotherapeutics. However, despite promising results in preclinical settings, the applicability of these agents to human has met with only limited success, largely due to inefficient systemic delivery and bioavailability. For example, the study of pharmacokinetics of resveratrol in humans concluded that even high doses of resveratrol might be insufficient to achieve resveratrol concentrations required for the systemic prevention of cancer [29]. Similarly, the amount of EGCG or curcumin required to observe anticancer effects in humans is too high to be feasibly incorporated in a clinical trial due to acute toxicity [30]. Therefore, to achieve maximum response of a chemopreventive agent, novel strategies are required to enhance the bioavailability of potentially useful agents and reduce perceived toxicity.

Nanomedical approaches to drug delivery center on developing nanoscale particles or molecules to improve drug bioavailability [31,32]. Drug delivery focuses on maximizing bioavailability both at specific places in the body and over a period of time. Numerous different strategies have been employed in nanotechnology to optimize drug delivery in a tumor specific manner. This technology extends to different disciplines of science such as chemistry, environmental science, tissue engineering, as well as in medicine where it is gradually cementing its position as a potent therapeutic option. Different approaches have been applied to effectively load target drugs to enhance delivery that are based on the solubility properties of drugs to be loaded and are discussed below.

## 2. Types of Nano Formulations

**Figure 1.**
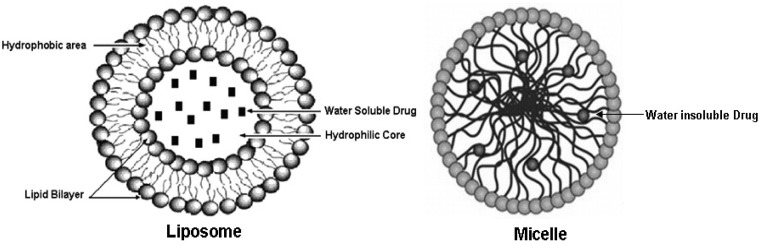
Two major loading models—Liposome and Micelle—for effectively loading water soluble and water insoluble natural chemopreventive agents, respectively. (Micelle image is a modified figure from Nature Reviews [43].

The choice of nano formulation to be used depends on the solubility of the drug of interest that has to be loaded. The two most popular and well-investigated drug carriers are liposomes (for the delivery of water-soluble drugs) and micelles (for the delivery of water insoluble drugs) (see Figure 1). Liposomes are artificial phospholipid vesicles that are usually less than 1,000 nm in size and can be loaded with a variety of water-soluble drugs that sit in its inner compartment. Without doubt these liposomes have been considered to be promising drug carriers [33,34]. They are biologically inert and completely biocompatible, and they cause practically no toxic or antigenic reactions; drugs included in liposomes are protected from the destructive action of external media [35]. The use of targeted liposomes (that is, liposomes selectively accumulating inside an affected organ or tissue) increases the efficacy of the liposomal drug and decreases the loss of liposomes and their contents in the reticuloendothelial system [36,37]. To obtain targeted liposomes, many protocols have been developed to bind corresponding targeting moieties, including antibodies, to the liposome surface without affecting the liposome integrity and antibody properties [38,39]. However, the approach with immunoliposomes may nevertheless be limited because of their short life in the circulation [40,41]. Dramatically better accumulation can be achieved if the circulation time of liposomes is extended, increasing the total quantity of immunoliposomes passing through the target and increasing their interactions with target antigens. This is why long-circulated (usually, coated with PEG, *i.e.*, PEGylated) liposomes have attracted so much attention over the last decade [42] and will be discussed in the forthcoming passages.

The development of drug nanocarriers for water insoluble drugs has been a huge task, particularly because large proportions of new drug candidates emerging from high-throughput drug screening initiatives such as chemopreventive agents are water-insoluble. The therapeutic application of hydrophobic, poorly water-soluble agents like curcumin, resveratrol, EGCG, *etc.*, is associated with some serious problems, since low water-solubility results in poor absorption and low bioavailability [44]. In addition, drug aggregation upon intravenous administration of poorly soluble drugs might lead to such severe toxicity. On the other hand, the hydrophobicity and low solubility in water appear to be intrinsic properties of many drugs, since it helps a drug molecule to penetrate a cell membrane and reach important intracellular targets. This is why micelles, including polymeric micelles, are another promising type of pharmaceutical carrier for such water-insoluble drugs [45]. Micelles are colloidal dispersions with a particle size between 5 nm and 100 nm. An important property of micelles is their ability to increase the solubility and bioavailability of poorly soluble pharmaceuticals. The use of certain special amphiphilic molecules as micelle building blocks can also extend the blood half-life upon intravenous administration. Because of their small size (<100 nm), micelles demonstrate spontaneous penetration into the body compartments with leaky vasculature. In the case of targeted micelles, local release of a free drug from micelles in the target organ should lead to increased efficacy of the drug, while the stability of the micelles *en route* to the target organ or tissue should contribute better drug solubility and toxicity reduction, because of less interaction with non target organs.

## 3. Nanochemoprevention

The origin of nano-encapsulation based chemoprevention approaches could be traced to Mukhtar and co-workers who also coined the term nanochemoprevention for the first time. This group utilized the multi-functionality of biodegradable polylactic acid (PLA)–polyethylene glycol (PEG) nanoparticles to incorporate the well recognized chemopreventive agent from green tea ‘EGCG’. This revolutionary strategy showed effective antitumor efficacy in a prostate model and most importantly the PLA/PLGA nanoparticles when injected systemically were rapidly cleared by the mononuclear phagocytic system by the process of endocytosis, thereby minimizing carrier-induced undesirable cytotoxicity [46].

Since this proof of concept by Mukhtar's group, the field has made tremendous advancements with numerous groups trying to emulate and optimize the PLG based nano strategies. This review summarizes the most up to date knowledge of the recent concepts in nanochemoprevention with emphasis on major chemopreventive agents that have been proven for their anticancer potential.

### 3.1. Nanocurcumin

Curcumin, or diferuloylmethane, is a yellow polyphenol extracted from the rhizome of turmeric (*Curcuma longa*), a plant grown in tropical Southeast Asia [47]. For centuries, turmeric has been used as a spice and coloring agent for food, as well as a therapeutic agent in traditional medicine. Enthusiasm for curcumin as an anti-cancer agent evolved based on the wealth of epidemiological evidence suggesting a correlation between dietary turmeric and low incidence of gastrointestinal mucosal cancers [48]. However, like other promising dietary chemopreventive agents, curcumin could not have a clinical impact due to its rapid degradation and poor bioavailability in biological systems [49]. Considerable investigations have been performed all around the globe in order to increase curcumin's bioavailability, systemic delivery and thereby it's anticancer potential. Numerous nano based attempts have been made and it has been shown that nano loaded curcumin has better bioavailability parameters and efficacy and are described below.

Earliest attempts on loading the curcumin molecule with nanoparticles came from Plianbangchang and group where curcuminoids loaded solid lipid nanoparticles (SLNs) were developed using a microemulsion technique at fixed temperatures [50]. In this study, the authors reported that variation in the amount of ingredients had profound effects on the curcuminoid loading capacity, the mean particle size, and size distribution. However, these nanoformulations were designed as anti-ageing moieties and were not studied for their anticancer potential. Following this, Maitra and group synthesized polymeric nanoparticle encapsulated formulation of curcumin “nanocurcumin” utilizing the micellar aggregates of cross-linked and random copolymers of *N*-isopropylacrylamide (NIPAAM), with *N*-vinyl-2-pyrrolidone (VP) and poly(ethyleneglycol)monoacrylate (PEG-A) [51]. Physico-chemical characterization of the polymeric nanoparticles by dynamic laser light scattering and transmission electron microscopy confirmed a narrow size distribution in the 50 nm range. This nanocurcumin, unlike free curcumin, was shown to readily disperse in aqueous media and demonstrated comparable *in vitro* therapeutic efficacy to free curcumin against a panel of human pancreatic cancer cell lines. A different approach was taken by Tsuchida and group where they loaded curcumin in a stable lipid based formulation with intentions of routing curcumin specifically to macrophages [52,53]. The authors elegantly demonstrated that these nanoparticulate formulations could deliver curcumin into tissue macrophages, specifically bone marrow and splenic macrophages in rats, and suggested that the intravenous delivery system of curcumin using lipid-based nanoparticles may be available for antioxidant and anti-inflammatory therapies. In another study, Sahu *et al.* synthesized a novel polymeric amphiphile, mPEG-PA, with methoxy poly(ethylene glycol) (mPEG) as the hydrophilic and palmitic acid (PA) as the hydrophobic segment. This mPEG-PA conjugate was shown to undergo self-assembly in an aqueous environment and could encapsulate highly hydrophobic compounds like curcumin in the nanocarrier, making the drug readily soluble in an aqueous system, which could increase the ease of dosing and make intravenous dosing possible [54]. Drug-loaded micelle nanoparticles showed good stability in physiological conditions; however, did not reduce the IC_50_ of curcumin.

Kumar *et al.* have worked on the development of curcumin nano based system and have shown curcumin encapsulated nanoparticles prepared by emulsion technique were able to withstand the International Conference on Harmonization (ICH) accelerated stability test conditions for refrigerated products for the studied duration of three months [55]. In their study, the *in vivo* pharmacokinetics revealed that curcumin entrapped nanoparticles demonstrate at least nine-fold increase in oral bioavailability when compared to curcumin administered with piperine as absorption enhancer. Similar work was done by Mulik *et al.*, where they created Poly(butyl) cyanoacrylate (PBCA) nanoparticles coated with poloxamer 188 containing curcuminoids by anionic polymerization using solvent evaporation method. *In vitro* release study showed that these prepared PBCA nanoparticles were capable of controlled drug release (following matrix model) for an extended period of time with higher release in acidic environment compared to PB 7.4 suggesting the usefulness of the prepared nanoparticles for intracellular delivery [56]. Other approaches include blending silk fibroin (SF) and chitosan (CS) polymers to encapsulate curcumin [57]. The uptake and efficacy of SF-coated curcumin was significantly higher than SFCS-coated curcumin in both low and high Her2/neu expressing breast cancer cells and was suggested as potential treatment in *in vivo* breast tumors by local, sustained, and long-term therapeutic delivery as a biodegradable system. While Lvov and group used layer by layer gelatin coated nanoparticles to efficiently deliver curcumin and other polyphenols in breast tumors [58]. Bora and group have reported a nanoformulation of curcumin with a tripolymeric composite for delivery to cancer cells. The composite nanoparticles (NPs) were prepared by using three biocompatible polymers: alginate (ALG), chitosan (CS), and pluronic-by ionotropic pre-gelation followed by polycationic cross-linking. Pluronic F127 was used to enhance the solubility of curcumin in the ALG-CS NPs. However, like other methodologies described above only the delivery of curcumin was benefited and not its potency [59]. Another important milestone was achieved by Aggarwal and group where they employed a polymer-based nanoparticle approach to improve bioavailability. In this study, curcumin was encapsulated with 97.5% efficiency in biodegradable nanoparticulate formulation based on poly (lactide-co-glycolide) (PLGA) and a stabilizer polyethylene glycol (PEG)-5000 [60]. The authors observed *in vitro* curcumin (NP) exhibited very rapid and more efficient cellular uptake than curcumin. The formulation had better apoptotic activity, greater inhibition of TNF-induced NF-kappaB activation and enhanced suppression of NF-kappaB-regulated proteins involved in cell proliferation (cyclin D1), invasion (MMP-9), and angiogenesis (VEGF). They also observed that in mice, curcumin (NP) was more bioavailable and had a longer half-life than curcumin.

In another important study, nanocrystal solid dispersion (CSD-Cur), amorphous solid dispersion (ASD-Cur), and nanoemulsion (NE-Cur), were designed with the aim of improving physicochemical and pharmacokinetic properties of curcumin [61]. In dissolution tests, all curcumin formulations exhibited marked improvement in the dissolution behavior when compared with crystalline curcumin. Significant improvement in pharmacokinetic behavior was observed in the newly developed formulations, as evidenced by increase of oral bioavailability. Mukherjee and Vishwanathan have shown efficiency of encapsulation of curcumin in poly (lactic-coglycolic acid) (PLGA) nanospheres using solid/oil/water emulsion solvent evaporation method [62]. They observed successful formation of smooth and spherical curcumin-loaded PLGA nanospheres, with an encapsulation efficiency of 90%. Evaluation of these curcumin-loaded nanospheres was carried out in prostate cancer cell lines. The authors showed robust intracellular uptake of the nanospheres in the cells. Cell viability studies revealed that the curcumin-loaded nanospheres were able to exert a more pronounced effect on the cancer cells as compared to free curcumin. It was concluded that such nanoparticle-based formulation of curcumin has high potential as an adjuvant therapy for clinical application in prostate cancer. In another study, PLGA nanoparticles encapsulating curcumin were synthesized and used to treat two different cystic fibrosis (CF) mouse strains in an effort to correct the defects associated with CF by improving bioavailability of the compound, which had previously been a challenge in treatment with curcumin. These studies revealed that oral administration of PLGA nanoparticles encapsulating curcumin enhances the effects of curcumin therapy in CF mice, as compared to delivery of nonencapsulated curcumin [63].

Progress has also been made in the synthesis of self-organized mixed assemblies of curcumin and a poly(oxyethylene) cholesteryl ether (PEG-Chol) [64]. Curcumin was assembled together with PEG-Chol to form nano-sized assemblies (around 10 nm) of assumed micelles that, in contrast with the rapid decomposition of free curcumin, the curcumin-PEG-Chol was highly stabilized in the nanoparticles. These studies showed that the cytotoxic activity of the curcumin/PEG-Chol nanoparticles against myeloma cells is higher than those of free curcumin. On the other hand, both the curcumin/PEG-Chol nanoparticles and PEG-Chol micelles had significant cytotoxicity to the myeloma cells at 5 μM. Another group has designed curcumin nanoparticles using Eudragit S100 polymer that dissolves at colonic pH to result in selective colonic release of the entrapped drug [65]. Solvent emulsion-evaporation technique was employed to formulate these nanoparticles. The obtained freeze-dried nanoparticles exhibited a negative surface charge, drug content of >99% and presence of drug in amorphous form which may result in possible enhanced absorption. Interestingly this formulation demonstrated almost double inhibition of the cancerous cells, as compared to curcumin alone, at the concentrations tested. Enhanced action may be attributed to size influenced improved cellular uptake, and may result in reduction of overall dose requirement giving clinical relevance to these studies. Yallapu *et al.* have shown that in resistant ovarian cancer cells, a nano formulation of curcumin induces chemo/radio sensitization and inhibits growth of these cells [66]. Although this was also a PEG based nano formulation, additional antibodies were, however, conjugated that increased its potency. A newer method of nanoparticle formulation for poorly water-soluble materials was demonstrated for curcumin by Lvov's group where the drug was dissolved in organic solvent that is miscible with water (ethanol), and drug nucleation was initiated by gradual worsening of the solution by the addition of an aqueous polyelectrolyte assisted by ultrasonication [67]. Using this newer approach, curcumin crystals of 60–100 nm size were obtained depending on the component concentrations, sonication power, and initial solvent. Layer-by-layer shell assembly with biocompatible polyelectrolytes was used to provide a particle coating with a high surface potential and the stabilization of drug nanocolloids. Polyelectrolyte layer-by-layer encapsulation allowed sustained drug release from nanoparticles over the range of 10–20 h. Very recently, as an improvement on the PEG nano formulation of curcumin, such formulations were made in the presence of poly(vinyl alcohol) and poly(L-lysine) stabilizers, using a nano-precipitation technique [68]. These curcumin nano-formulations were characterized for particle size, zeta potential, drug encapsulation, drug compatibility and drug release. Encapsulated curcumin existed in a highly dispersed state in the PLGA core of the nanoparticles and exhibited good solid-solid compatibility. An optimized curcumin nano-formulation (nano-CUR6) has demonstrated two- and six-fold increases in the cellular uptake performed in cisplatin resistant A2780CP ovarian and metastatic MDA-MB-231 breast cancer cells, respectively, compared to free curcumin. In these cells, nano-CUR6 has shown an improved anti-cancer potential in cell proliferation and clonogenic assays compared to free curcumin.

The last decade has witnessed a huge progress on nano based enhancement of the anticancer potential of curcumin. The nano alterations to curcumin have been more focused on enhancing its bioavailability rather than its therapeutic activity. However, it should be noted that the field is still progressing and needs a lot of improvement in order to achieve clinically beneficial results. Nevertheless, initial studies mentioned in the preceding text certainly point to the proof of concept of this technology and one may see such formulations in the clinic soon.

### 3.2. Nanoresveratrol

Resveratrol (3,5,4′-trihydroxy-trans-stilbene) is a phytoalexin produced naturally by several plants when under attack by pathogens such as bacteria or fungi. Resveratrol and its effects is currently a topic of numerous animal and human studies. In mouse and rat experiments, anti-cancer [69], anti-inflammatory, blood-sugar-lowering and other beneficial cardiovascular effects of resveratrol have been reported. However, most of these results have yet to be replicated in humans. As with other natural chemopreventive agents, resveratrol also has a very short half life and is rapidly glucoronated and sulfonated, aiding its rapid turnover and excretion. Therefore, researchers focused on ways to enhance the bioavailability of resveratrol by different approaches and nano based studies were among the major driving force in this area.

The earliest reported nano formulation of resveratrol comes from a study by Yao *et al.*, where they prepared resveratrol chitosan nanoparticles with free amine groups on the surface so as to conjugate ligands, which will actively target to special tissues or organs [70]. These chitosan nanoparticles with free amines on the surface were obtained by sodium chloride precipitation through which nanoparticles with different solidification degrees were studied on turbidity, *in vitro* release, encapsulation efficiency, drug loading and diameter. Another liposomal nano based approach was done by Wang and group, where they showed that resveratrol release from nanoliposomes *in vitro* fitted the log-normal distribution equation and had a property of sustained release [71]. These studies concluded that Res-nanoliposomes could sustain to release drug *in vitro*. Interestingly, the absorption was a first-order process with the passive diffusion mechanism and also supported that notion that Res-nanoliposomes could promote the absorption of Res in rat small intestine. In one of the most clinically relevant study by Shao and co-workers, resveratrol was incorporated into mPEG-PCL based nanoparticles with high encapsulation efficiency due to its lipophilicity that resulted in significantly higher efficacy compared to equivalent dose of free Res [72]. Furthermore, free Res showed significant less cytotoxicity than the equivalent dose of Res-loaded nanoparticles with the preconditioning of Vitamin E. Meanwhile, reactive oxygen species (ROS) determination revealed significantly lower intracellular ROS levels in Res-treated cells compared to nanoparticle-treated cells. The authors suggested that the differential cytotoxicity between Res and Res-loaded nanoparticles may be mediated by the discrepancy of intracellular ROS levels. Recently resveratrol-loaded polymeric micelles were shown to protect from abeta-induced ROS generation [73]. In another study, solid lipid nanoparticles (SLN) were used as a carrier for resveratrol. The effects of SLN—empty or loaded with resveratrol (SLN-RSV)—on the internalization, growth, morphology, metabolic activity and genetic material of keratinocytes were compared to those of resveratrol in solution [74]. The cytostatic effect of SLN-RSV was much more expressed than that of RSV in solution and the authors proposed that delivery of RSV by SLN contributes to effectiveness of RSV on decreasing cell proliferation and has potential benefits for prevention of skin cancer. Very recently studies were done by Keck and group to develop resveratrol nano suspensions for dermal application. In this study, four nanosuspensions were investigated using the stabilizers Tween 80, Poloxamer 188, Plantacare 2000 and Inutec SP1, 1% and 2%, respectively. The plantacare and Inutec showed best stabilization that was attributed solely to steric stabilization [75].

### 3.3. Epigallocatechin Gallate EGCG Nano Formulation

The idea that tea polyphenols, especially active component EGCG, could have chemopreventive properties came from seminal studies by Mukhtar and group more than a decade ago [76]. Since then, tremendous research effort has been made to decipher the mechanism behind such observed anti-neoplastic effects. Despite promising results in preclinical settings, its clinical applicability to humans has met with limited success largely due to inefficient systemic delivery and bioavailability. In the first study of its kind, Mukhtar and group introduced the concept of nanochemoprevention, which uses nanotechnology for enhancing the outcome of chemoprevention [77]. In this study, the authors encapsulated green tea polyphenol EGCG in polylactic acid-polyethylene glycol nanoparticles and observed that encapsulated EGCG retained its biological effectiveness with over 10-fold dose advantage for exerting its proapoptotic and angiogenesis inhibitory effects, critically important determinants of chemopreventive effects of EGCG in both *in vitro* and *in vivo* systems. This important study paved the way for the use of nanoparticle-mediated delivery to enhance bioavailability and limit any unwanted toxicity of chemopreventive agents, such as EGCG. Monfilliette-Dupont and group used encapsulation method to enhance the stability of EGCG and related cathecins and with simple chemical modification of EGCG, it was possible to reach very high encapsulation rates (95%). Stable colloidal suspensions of (-)-EGCG were obtained in water over four weeks while free (-)-EGCG solubilized in water exhibited 100% degradation within four hours [78].

Lvov and group, who had earlier worked on curcumin nanoformulations, also applied their technology in developing EGCG based nano therapies. In this study, EGCG, tannic acid, curcumin, and theaflavin were encased into gelatin-based 200 nm nanoparticles consisting of a soft gel-like interior with or without a surrounding LbL shell of polyelectrolytes (polystyrene sulfonate/polyallylamine hydrochloride, polyglutamic acid/poly-l-lysine, dextran sulfate/protamine sulfate, carboxymethyl cellulose/gelatin, type A) assembled using the layer-by-layer technique [79,80]. Interestingly nanoparticle-encapsulated EGCG retained its biological activity and blocked hepatocyte growth factor (HGF)-induced intracellular signaling in the breast cancer cell line MBA-MD-231 as potently as free EGCG. In another study from China, folate mediated EGCG bovine serum albumin nanoparticles (FA-EGCG-BSANP) were prepared by desolvation process [81]. FA-EGCG-BSANP could significantly promote EGCG to PC-3 cell sites and improve their efficacy. Although not in the context of cancer yet, Smith and group observed that nanolipidic particles improve the bioavailability and alpha-secretase inducing ability of EGCG for the treatment of Alzheimer's disease [82]. Recently, Larson and co-workers have shown that chitosan nanoparticles enhance the intestinal absorption of the green tea catechins (+)-catechin and (-)-epigallocatechin gallate [83]. Progress on the development of EGCG nano formulations has been slow and is still in its infancy.

### 3.4. Genistein Nanoencapsulation

Genistein, a soy derived isoflavone, has recently attracted much attention of the medical scientific community. This compound was found to be a potent agent in both prophylaxis and treatment of cancer as well as other chronic diseases [84,85]. The great interest that has focused on genistein led to the identification of numerous intracellular targets of its action in the live cell [86]. At the molecular level, genistein inhibits the activity of ATP utilizing enzymes such as: tyrosine-specific protein kinases, topoisomerase II and enzymes involved in phosphatidylinositol turnover [87]. Moreover, genistein can act via an estrogen receptor-mediated mechanism [88]. At the level one step higher, *i.e.*, at the cellular level, genistein induces apoptosis and differentiation in cancer cells, inhibits cell proliferation, modulates cell cycling, exerts antioxidant and prooxidant effects, inhibits angiogenesis, and suppresses osteoclast and lymphocyte functions [89,90]. These activities make genistein a promising innovative agent in the treatment of cancer.

Although genistein has been shown to possess anticancer activities in different experimental systems, yet the same effects could not be translated in the clinical setting due to its poor bioavailability. Newer formulations of genistein such as diindolylmethane (B-DIM) from Bioreseponse Inc. has shown some enhanced bioavailability [91]. Researcher have tried various nano approaches including incorporation of genistein into topical nanoemulsion formulations composed of egg lecithin, medium chain triglycerides (MCT) or octyldodecanol (ODD) and water by spontaneous emulsification [92]. Poli and group have designed numerous flavonoid nano formulations over the last few years. They have extensively reviewed their studies in a seminal article where they have shown that incorporation into lipidic or polymer-based nanoparticles appears to markedly help the oral delivery of flavonoids, as these particles can protect the drug from degradation in the gastrointestinal tract and, by virtue of their unique absorption mechanism through the lymphatic system, also from first-pass metabolism in the liver [93]. Another approach was undertaken by Zhao and co-workers which was based on covalently attaching genistein onto Fe_3_O_4_ nanoparticles coated by cross-linked carboxymethylated chitosan (CMCH) [94]. The effects of free genistein and FeO_4_-CMCH-genistein nano-conjugate on the proliferation and apoptosis of gastric cancer cell line SGC-7901 were investigated and the results indicated that the Fe_3_O_4_-CMCH-genistein nano-conjugate exhibits a significantly enhanced inhibition effect to the SGC-7901 cancer cells than the free genistein. This type of drug delivery system is certainly promising for future multifunctional chemotherapeutic application as it combines drug release and magnetic hyperthermia therapy.

### 3.5. Nano Formulations of Taxol (Paclitaxel)

As mentioned previously, Taxol is among the first clinically and FDA approved chemotherapy drug that originated from natural sources. Its brand name Paclitaxel is a mitotic inhibitor used in cancer chemotherapy [95]. It was discovered from the bark of the Pacific yew tree, *Taxus brevifolia*, and thus named taxol [96,97]. Paclitaxel is water insoluble and is dissolved in Cremophor EL and ethanol, as a delivery agent. A newer formulation, in which paclitaxel is bound to albumin, is sold under the trademark Abraxane [98]. In order to overcome solubility issues, its first nanoformulation was developed by Onyuksel and group using sterically stabilized phospholipid micelles (SSMs) composed of poly(ethylene glycol-2000)-grafted distearoyl phosphatidylethanolamine (PEG(2000)-DSPE) as new lipid-based carriers for water-insoluble drugs [99]. In their initial report the group compared sterically stabilized mixed micelles (SSMM), composed of (PEG(2000)-DSPE) plus egg-phosphatidylcholine, with SSM as a novel delivery system for improved solubilization of water-insoluble drugs using paclitaxel as a model [100]. The authors concluded that SSMM showed increased solubilization potential compared with SSM while retaining all of its own advantages and proved to be an excellent technology for water-insoluble drugs.

The same group improved on their SSM based technology by stabilizing, biocompatible and biodegradable sterically stabilized mixed phospholipid nanomicelles (SSMM; size, approximately 15 nm) to actively targeted vasoactive intestinal peptide-grafted SSMM (SSMM-VIP) [101]. Paclitaxel loaded in SSMM (P-SSMM) and SSMM-VIP (P-SSMM-VIP) significantly inhibited cell growth in a dose-dependent fashion (p < 0.05). Both formulations were approximately seven-fold more potent than paclitaxel dissolved in DMSO (P-DMSO). Efficacy of P-SSMM and P-SSMM-VIP was similar (p > 0.5). The P-SSMM-VIP showed superior activity against drug resistant BC19/3 cells breast cancer cells compared to P-SSMM or P-DMSO, approximately two- and five-fold, respectively; p < 0.05. Collectively, their studies indicated that actively targeted paclitaxel-loaded SSMM-VIP overcomes multiple drug resistance of breast cancer cells and suggested that this formulation should be further developed to treat MDR breast cancer.

## 4. Conclusions

Nanotechnology has cemented its position in the area of chemoprevention where its finds wide ranging applications. Researchers are now able to effectively utilize nanoscale technologies to circumvent the issues of poor bioavailability and solubility associated with natural dietary agents. Rapid advancements have been made in this area on improving the nano formulations of various chemopreventive agents such as curcumin, resveratrol, genistein, *etc.* Work has also been done to load multiple anticancer agents with hope to create one magic bullet for the effective treatment of different cancers. Similarly, progress is also being made in the development of tumor targeted natural agent drug delivery systems that incorporate systems against specific recognition sequence such as VIPs. However, much needs to be done before we have an ideal carrier for these promising anticancer and chemopreventive agents. Nevertheless, based on the important progress made in the science of nanochemoprevention, it is suggested that this strategy should be further explored for its potential use in both the preclinical and clinical setting. With evident laboratory achievements in this technology all across the globe, one can expect the appearance of “real” nanochemopreventive medicines in the not-too-distant future.
